# Stress reactivity to an electronic version of the Trier Social Stress Test: a pilot study

**DOI:** 10.3389/fpsyg.2015.00724

**Published:** 2015-05-29

**Authors:** Sage E. Hawn, Lisa Paul, Suzanne Thomas, Stephanie Miller, Ananda B. Amstadter

**Affiliations:** ^1^Department of Psychiatry, Virginia Institute of Psychiatric and Behavioral Genetics, Virginia Commonwealth University, Richmond, VA, USA; ^2^Department of Psychology, Northern Illinois University, DeKalb, IL, USA; ^3^Department of Psychiatry and Behavioral Sciences, Medical University of South Carolina, Charleston, SC, USA; ^4^School of Social Work, Virginia Commonwealth University, Richmond, VA, USA

**Keywords:** stress reactivity, Trier Social Stress Test, stressor, virtual reality, cortisol, TSST

## Abstract

Social stressors that rely on the inclusion of confederates (i.e., Trier Social Stress Test, TSST) are often used in clinical laboratory research paradigms to elicit a measurable stress response in participants. Although effective, the TSST is labor intensive and may introduce error variance as a function of confederate race, gender, and/or response characteristics. The present study aimed to develop and validate an electronic version of the TSST (e-TSST). The primary aim was to compare the e-TSST to an e-neutral control condition; the exploratory aim was to compare the magnitude of stress response elicited by the e-TSST to that elicited by the traditional TSST. Forty-three healthy adults were randomized to the e-TSST or e-neutral condition. Subjective (participant-rated distress) and objective [cortisol, heart rate (HR), and blood pressure] indices of stress were collected prior to, and multiple times following, the stressor. Using archival data collected from 19 healthy participants exposed to the traditional TSST in a prior study, stress reactivity was compared between the electronic and traditional versions of the TSST. The e-TSST elicited significant increases in all measures of stress reactivity compared to the e-neutral condition, with the exception of HR. Results showed that the magnitude of subjective distress, BP, and HR responses elicited by the e-TSST did not differ significantly from that elicited by the traditional TSST. The traditional TSST elicited significantly higher cortisol than the e-TSST. Although these findings provide initial support for the development of electronic versions of the TSST, further refinement of the e-TSST is warranted prior to broad adoption of this technology. A refined, reliable e-TSST could allow for increased utilization of the TSST by enhancing convenience, reducing labor costs, and limiting potential error variance introduced by human confederates.

## Introduction

### Importance of Studying Stress

It is widely accepted that stress impacts individuals psychologically and biologically. Stress is shown to negatively affect cognition and hypothalamus-pituitary-adrenal (HPA) axis regulation (Gerra et al., 2000; Lupien et al., 2009), perhaps contributing to documented relations between stress and mental health phenotypes, such as anxiety and mood disorders (Sapolsky, 1998). Additionally, many theories of addiction (e.g., tension reduction, stress-coping) implicate stress as an important trigger for substance use, craving, and relapse (Khantzian, 1985; Koob and Le Moal, 2001). Therefore, it is necessary that reliable and valid manipulations of the stress response are designed and utilized in order for stress to be studied more effectively. There are existing paradigms, such as the Trier Social Stress Test (TSST), described below, that produce subjective as well as physiological reactions to stress within a controlled laboratory setting (Kirschbaum et al., 1993). However, these paradigms are prone to environmental confounds (e.g., performance of confederates) and developing more convenient, efficient versions of this paradigm would make it easier to study participants’ stress responses. The use of new technology, like virtual reality (VR), may be able to meet these goals by limiting potential confounds and adding to the convenience and reliability of measuring stress in a controlled laboratory setting. Therefore, we sought to develop and validate a VR (i.e., electronic) version of the TSST (e-TSST).

### TSST Paradigm

The TSST, used regularly by researchers around the world, is supported as a “gold standard” human laboratory-based assessment of neuroendocrinological and psychological stress reactivity (Kirschbaum et al., 1993; Dickerson and Kemeny, 2002). The paradigm requires participants to perform a stress-inducing task (i.e., a speech and a mental math task) in front of three confederates who are instructed to maintain neutral expressions throughout the experiment. One drawback of the TSST is that it is necessary to gather at least three confederates to serve as stone-faced audience members, which is time consuming and can result in potential variability among audiences (e.g., effects may arise by confederates’ gender, race/ethnicity, age). Further, confederates’ reactions are critical, as any unstandardized indication of (dis)approval (e.g., nodding, frowning) can lead to changes in stress reactivity, and therefore introduce a source of error (Bruehl and Solar, 1970).

### VR Background

Virtual reality technology has recently gained popularity for use by clinicians conducting exposure-based therapies for anxiety disorders such as posttraumatic stress disorder, specific phobias, and more recently, social phobia. A sense of immersion into a virtual environment has proven an effective tool for studying a number of psychological phenotypes, as shown by previous research. Multiple studies demonstrate that VR paradigms can elicit anger and fear responses (e.g., Rothbaum et al., 2000; Macedonio et al., 2007). Results from over 13 randomized clinical trials were published comparing VR to *in vivo* exposure (the first line behavioral treatment for anxiety disorders in which the patient is repeatedly exposed to the feared stimulus until extinction of the feared response occurs). This approach is used for a variety of anxiety disorders, for example, acrophobia (Emmelkamp et al., 2002) and for the fear of flying (Rothbaum et al., 2000, 2006), and each showed that VR exposure was as effective as standard *in vivo* exposure in reducing anxiety. In a recent meta-analysis VR anxiety disorder therapy had a large effect size (*d* = 1.11) and was slightly more effective than *in vivo* therapy (Powers and Emmelkamp, 2008).

There is a growing body of literature suggesting that VR-based stress induction tasks can elicit psychophysiological stress responses in non-clinical samples, as well. For example, Bullinger et al. (2005) reported increased cortisol secretion in subjects performing a cognitive stress task in a dynamic VR environment compared to those exposed to a static VR environment (no-stress) task. Grillon et al. (2006) also identified robust physiological [i.e., heart rate (HR), skin conductance] responses to giving a speech in a VR environment.

### VR TSST

Wedding VR technologies to the standardized TSST model, if effective, has the potential to revolutionize the way in which stress reactivity is elicited by researchers. Jönsson et al. (2010) used mounted headgear to immerse participants into their VR version of the TSST. They found that this virtual TSST evoked significant increases in cortisol and HR at the first stress provocation when compared to a neutral condition, and that these responses were comparable to responses elicited from previous studies examining the effects of the live TSST. Jönsson et al. (2010) used animated confederates instead of live confederates, leaving a need to examine the efficacy of an electronic TSST that uses pre-recorded live actors, which might increase believability and, thus, be more comparable to the live TSST. Similarly, Wallergård et al. (2011) used an immersion headset to examine the efficacy of the VR-TSST in eliciting stress in a sample of seven healthy males. Physiological study outcomes were limited to assessments of HR (i.e., high frequency HR variability, T-wave amplitude, and HR). Furthermore, Wallergård et al. (2011) also used animated confederates in their VR-TSST simulation. These limitations maintain a gap in the literature, which calls for the need to examine a broader array of physiological and neuroendocrine stress responses to a virtual TSST, as well as a need to examine the efficacy of using live actors within an electronic medium.

In another study comparing neuroendocrine reactivity to traditional, virtual (mounted headgear), and imaginary (one-way mirror) administrations of the TSST, Kelly et al. (2007) found that, although the virtual TSST evoked cortisol increases comparable to the imagined audience, it was less effective than the traditional TSST. Although Kelly et al. (2007) used pre-recorded actors in their virtual TSST, the actors were superimposed into the virtual interview room, leaving a need to examine the efficacy of an electronic TSST that uses pre-recorded live actors, taped in their live environment, to increase believability and create an electronic TSST more comparable to the live TSST. Notably, Kelly et al. (2007) compared cortisol levels elicited by the TSST modalities only, such that no comparisons between each modality and a control (or neutral condition) were made. It is important to note that each of the three existing studies examining a VR-TSST used immersion headset technology, which requires the purchase and storage of expensive and burdensome equipment. Additionally, although Kelly et al. (2007) used live confederates, none of the existing limited number of studies examined the use of TSST in a non-avatar environment. Furthermore, although Kelly et al. (2007) compared their VR-TSST to a live version, they did not compare any of the paradigms to a neutral control.

With regard to the induction of a measureable stress response, the virtual audience reduces the burden on the researcher for gathering and organizing audience participants and eliminates potential confounds resulting from confederate variation in audience demographics and response to the participant. To this end, the present study sought to create an e-TSST paradigm, and to examine if the e-TSST paradigm could elicit a stress response in healthy participants. Thus, this study helps to build upon the growing body of literature showing that VR paradigms are as effective as *in vivo* exposure in eliciting anger and fear, psychophysiological stress, and other anxiety-related responses (Rothbaum et al., 2000, 2006; Emmelkamp et al., 2002; Bullinger et al., 2005; Grillon et al., 2006; Macedonio et al., 2007; Powers and Emmelkamp, 2008). To our knowledge, this is the first study to date not only to examine the efficacy of an electronic TSST, administered in a non-avatar environment, to a neutral electronic condition, but also to compare it to a live “gold-standard” TSST. Therefore, this study addresses some gaps in the current literature by implementing methods that will increase believability (i.e., live actors recorded in a live and realistic environment) and accessibility (i.e., low-cost equipment), thereby providing an innovative, standardized laboratory methodology for stress induction.

### Aims and Hypotheses

Collaborating with Virtually Better, Inc., an Atlanta, Georgia based company known internationally as a leader in developing virtual reality systems for behavioral health assessment and treatment applications, we created the e-TSST to test the hypothesis that the e-TSST would elicit a stress response in healthy participants compared to a neutral electronic (e-neutral) condition (i.e., looking at a virtual reality aquarium). An exploratory hypothesis was that the magnitude of the stress response elicited by the e-TSST would not differ from that elicited by the traditional TSST (using archival data from our previous study utilizing the TSST). Stress reactivity was assessed using both subjective and objective measures of stress reactivity [i.e., subjective distress, serum cortisol, HR, and blood pressure (BP)].

## Materials and Methods

### Overview

The study was a between-subjects design in which participants were randomized to the electronic stress (e-TSST) or no-stress (e-neutral) condition. Individuals meeting basic eligibility criteria completed an office visit assessment, and those meeting final eligibility criteria were brought to the Clinical and Translational Research Center (CTRC). Baseline and post-stress physiological and subjective indices of stress were collected. Data from the e-TSST condition was compared to the traditional TSST using archival data. The Medical University of South Carolina Institutional Review Board approved all study procedures and informed consent was obtained from all study participants.

### Participants

Participants in both study samples (e-TSST and traditional TSST) were recruited through the community by advertising (e.g., newspaper, flyers, internet), and through collaborations with other researchers using healthy control participants. Inclusion criteria were an age between 21 and 65 years old and the ability to provide informed consent. Exclusion criteria were the presence of a condition that affected HPA axis functioning (e.g., individuals taking psychoactive medications, antihistamines, or anti-inflammatory medications, alcohol dependence; a current major Axis I disorder; hypertension, chronic pain, Addison’s disease) and factors that would affect stress or stress hormones (e.g., smokers who could not abstain from smoking for at least 4 h, severe obesity [i.e., BMI ≥ 40]). Individuals with any blood clotting disorder were also excluded due to the required blood draw. Forty-three participants total were included in the electronic sample (e-TSST vs. e-neutral). The subsample in the e-TSST condition were compared to archival data from 19 participants who completed the traditional TSST.

Participant characteristics are shown in Table 1. The mean age of participants in the e-TSST condition was 37.5 (SD = 12.5), and the majority of participants were Caucasian (79.1%), and 48.8% were female. Nineteen participants (*M* age = 30.3 years, SD = 11.0, 89.5% Caucasian, 52.6% female) total were included in the traditional TSST group. The mean age of participants in the e-TSST condition is slightly higher than the mean age demonstrated in previous TSST studies (e.g., mean ages of 28.3, 22.4, 22.6, and 21.46; Kirschbaum et al., 1992; Kelly et al., 2007; Jönsson et al., 2010, respectively).

**Table 1:** **Descriptive statistics by study sample**.

	**Electronic TSST *n* = 43**		**Traditional TSST *n* = 19**
	**e-Neutral**	**e-TSST**	**TSST**
Female (%)	52.4	45.5	52.6
Caucasian (%)	85.7	72.7	89.5
African American (%)	14.3	13.6	10.5
Age, *M* (SD)	36.5 (13.22)	38.45 (12.00)	30.32 (11.04)
Anxiety sensitivity, *M* (SD)	26.57 (5.84)	26.59 (6.31)	15.42 (9.12)
Beck depression, *M* (SD)	0.43 (1.54)	0.23 (0.61)	3.11 (3.18)
Social phobia, *M* (SD)	7.90 (6.08)	9.95 (9.23)	12.42 (9.31)
State anxiety, *M* (SD)	30.10 (8.92)	31.20 (7.25)	32.21 (7.75)

Participants assigned to the e-neutral vs. e-TSST conditions did not differ from each other on any variables.

### Procedure

#### Office Session

Potential participants underwent screening via telephone or electronic survey, and those meeting preliminary criteria came in for an office visit, beginning at 3:00 pm. Participants were instructed to not consume caffeine and to not eat after noon on the day of the study, as both food and caffeine can introduce noise variability to neuroendocrine reactivity. The office visit included the provision of informed consent, and the completion of self-report measures and a structured clinical interview to confirm eligibility.

All participants were assessed with validated instruments to diagnose psychiatric disorders and to assess the severity of anxiety and depression symptoms. Specifically, the Mini-International Neuropsychiatric Interview (MINI; Sheehan et al., 1998) was used to rule out the presence of exclusionary major Axis I disorders. Anxiety symptoms were assessed with the following instruments: State-Trait Anxiety Inventory (ranging 20–80; Spielberger et al., 1969) to assess state anxiety [alpha = 0.91]; the Anxiety Sensitivity Index-3 (ASI-3; Taylor et al., 2007), ranging 0–72, to assess propensity to interpret feelings of anxiety as catastrophic [alpha = 0.79]; and the Social Phobia Anxiety Inventory (SPAI; Turner et al., 1989), ranging 0–192, to assess severity of social anxiety symptoms [alpha = 0.89]. Depression symptoms were measured with the Beck Depression Inventory II (BDI-II; Beck et al., 1996) [alpha = 0.68], ranging 0–63.

#### Challenge Session Procedures

Following the office visit, participants who did not meet criteria were compensated for their time. Eligible participants were randomized to the stress (i.e., e-TSST) or no-stress (i.e., e-neutral) condition by sex. These participants were taken to the CTRC at 4:00 pm to begin testing. The participant was fitted with an indwelling catheter to facilitate blood draws (which were assayed for cortisol). The experimental stress challenge (i.e., e-TSST or e-neutral procedure) began at 5:00 pm and assessments were collected every 15 min following the challenge (5:15, 5:30, 5:45, and 6:15).

**FIGURE 1:**
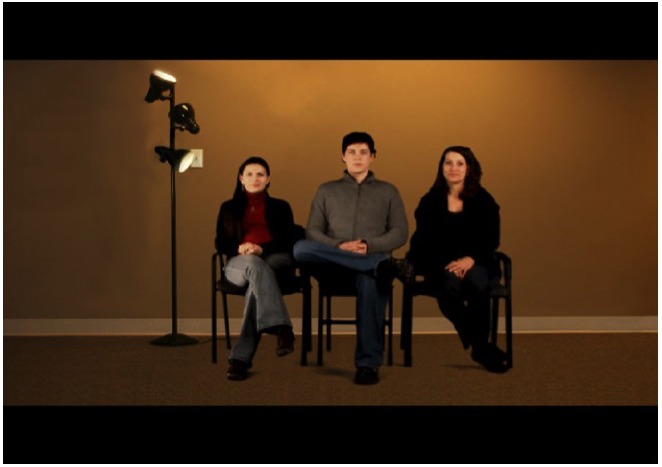
**Stress reactivity over the post-assessment time points for each index of stress in the e-TSST conditions.** Asterisk indicates a significant group difference in the follow-up contrast test.

Participants in the e-neutral condition viewed a virtual aquarium on a television screen for 15 min. Participants randomized to the e-TSST group were told that they would soon speak to an audience via closed-circuit video on why they should be hired for a particular job (the participant’s “dream job”). The participant was instructed that live audience members would be conferenced in, and that the presentation should be made to the television screen on the wall as though speaking in-person to a group of hiring managers. Participants were told that they had 5 min to prepare the speech, and the experimenter started the countdown clock (which was placed in view of the individual) and left the room. At 5:05 pm, the experimenter entered the room and told the participant to stand in front of the television screen for the speech; all of the participants’ notes were removed. Participants’ view of the e-TSST condition is shown in Figure 1. On the screen, the participant saw two women and one man seated in a tan room, empty with the exception of the confederates’ chairs and one lit lamp. The man, serving as the “spokesperson,” was seated between the two women. All confederates were Caucasian. Via the electronic environment, the spokesperson—operated by the experimenter who sat behind the participant and used keystrokes to control the e-TSST program—instructed the participant to stand and begin the prepared speech. The participant spoke for 5 min; any pauses were responded to by the virtual spokesperson with “Your time is not up, please continue” via the experimenter’s keystroke. At the end of the 5 min (5:10 pm), the virtual spokesperson—via a keystroke—instructed the participant to perform serial subtraction as quickly and accurately as possible for 5 min. Any errors were responded to by the virtual spokesperson with “Please begin again at the top” via a keystroke. Following this task, a keystroke was used to end the experiment and the virtual spokesperson said “The interview is now over; you may be seated.”

Similar to the live TSST, the e-TSST lasted 15 min. The first post-stressor assessment occurred immediately following the task (or e-neutral condition) at 5:15. A 2 ml blood draw was conducted using the in-dwelling catheter to obtain a serum cortisol assay to determine stress reactivity at each of the assessment time-points. Blood samples for cortisol were collected in iced EDTA tubes; plasma was separated from cells by centrifugation, and the serum sample was frozen at –70°C until thawed for assay. Cortisol was assayed using the ADVIA Centaur XP immunoassay system (Siemens Healthcare Diagnostics, Flanders, NJ, USA). Functional sensitivity was 0.2 g/dl, and intra-assay cv was 2.15% at 44 g/dl. Subjective units of distress (SUDs, range = 0–10, with 0 = No Anxiety, 10 = Extreme Anxiety), systolic BP, and HR were also assessed at these time points. Additional assessments occurred at 15, 30, and 60 min following the end of the stressor. At 6:15 pm, participants were debriefed, compensated for their time, and dismissed.

#### Traditional TSST Procedures

To best compare stress responses induced by e-TSST to those induced by the traditional TSST, we modeled the aforementioned procedures after those used in the archival study, using the traditional TSST paradigm. The same time points outlined above were followed in both sessions, as well as the randomization procedures used and the measurements administered. Due to budgetary differences across the studies, the archival sample had more assessment time-points than the electronic study; thus, only the assessments that aligned on time were used in the comparison analyses. Participants in the stress condition performed their 15-min task in front of live confederates, who were asked to maintain neutral expressions. Whereas the e-TSST “spokesperson” was manipulated to speak via programmed keystrokes, a delegated spokesperson in the standard TSST had received training to provide these same prompts in response to certain participant behavior (e.g., stopping the speech prior to the 5 min requirement).

### Data Analyses

First, all data was cleaned and one outlier was removed due to a score of six standard deviations above the mean (which exceeded our cutoff of greater than two standard deviations above the mean). To test the primary hypothesis, the e-TSST vs. e-neutral conditions were compared with a series of repeated measures ANCOVAs, one for each index of stress (SUDs, cortisol, HR, and systolic BP). The baseline value of each index of stress was employed as a covariate; the four post-stress assessments were the repeated measures, and condition (e-TSST vs. e-neutral) was the between-subjects variable. To test the exploratory hypothesis, another series of similar repeated measures ANCOVAs were conducted with e-TSST vs. traditional TSST as the between subjects variable. Again, the baseline assessment of each index of stress was employed as a covariate. Age, ASI-3 and BDI scores were also used as covariates, as they were significantly different between the study samples. A statistical correction was employed (Huyn-Feldt epsilon) if the assumption of sphericity was not met in the repeated measures analyses. Lastly, follow-up contrasts were conducted to determine the post-stress time-points that differed between conditions in both series of ANCOVAs.

## Results

### e-TSST Condition vs. e-Neutral Condition

**FIGURE 2:**
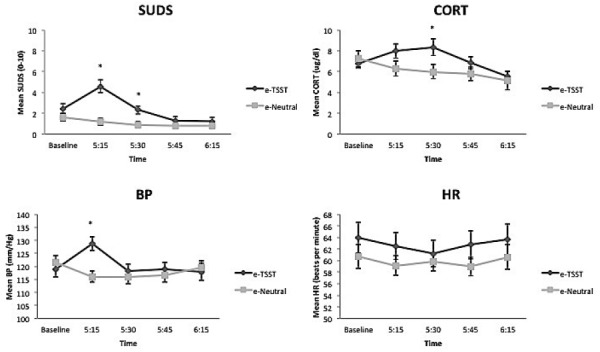
**Stress reactivity over the post-assessment time points for each index of stress in the e-TSST vs. traditional TSST conditions.** Asterisk indicates a significant group difference in the follow-up contrast test.

Repeated measures ANCOVAs demonstrated that the e-TSST condition evoked significant increases among all indices of stress (i.e., SUDs, cortisol, BP), when compared to the e-neutral condition, with the exception of HR. As shown in Figure 2, participants in the e-TSST condition responded to the experimental challenge with elevated SUDs [*F*(1.67,65.27) = 13.09, *p* < 0.001, ${\text{η}}_{p}^{2}$ = 0.25], cortisol [*F*(2.02,72.56) = 4.56, *p* < 0.05, ${\text{η}}_{p}^{2}$ = 0.11], and BP [*F*(3,108) = 12.37, *p* < 0.001, ${\text{η}}_{p}^{2}$ = 0.26], whereas the e-neutral participants’ SUDs remained relatively constant across the session, their cortisol steadily decreased, and their BP decreased slightly and then remained relatively constant. HR did not significantly differ between the conditions, *F*(2.92,113.84) = 1.01, *p* = 0.39, ${\text{η}}_{p}^{2}$ = 0.03. Follow-up contrasts revealed that the e-TSST group had higher SUDs compared to the e-neutral group at the first two post-task assessments (5:15 and 5:30), *t*s(41) = –4.74, –2.85, respectively, *p*’s < 0.01. Immediately post-stressor, there was a trend toward significance for the e-TSST group to have higher cortisol response than the e-neutral group, *t*(40) = –1.71, *p* = 0.095, that became significant at the second post-stressor assessment, *t*(40) = –2.28, *p* < 0.05, followed by a return to no significant difference between groups at the remaining time points. The e-TSST group had higher systolic BP than the e-neutral group at the first post-task assessment (5:15), *t*(41) = –3.64, *p*’s < 0.001 only.

### e-TSST Condition vs. Traditional TSST Condition

**FIGURE 3:**
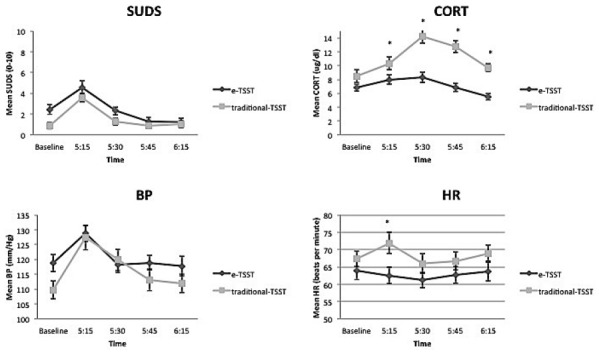
**e-TSST condition**.

Participants in the traditional TSST condition compared to the e-TSST condition were significantly younger [*F*(39) = 5.05, *p* < 0.05], and had lower ASI scores and higher BDI scores [*F*s(39) = 21.24,17.37, respectively, *p*’s < 0.001]; therefore, these variables were used as covariates in analyses that compared the TSST reactivity between the e-TSST and traditional TSST. Repeated measures analyses (ANCOVAs, covarying for baseline stress reactivity, age, BDI and ASI scores) were conducted to compare the two study samples. Stress reactivity in response to the TSST did not significantly differ across modalities (e-TSST vs. traditional) for SUDS [*F*(1.50,51.11) = 0.36, *p* = 0.64, ${\text{η}}_{p}^{2}$ = 0.01], BP [*F*(3,90) = 1.83, *p* = 0.15, ${\text{η}}_{p}^{2}$ = 0.06], or HR [*F*(2.62,89.10) = 0.91, *p* = 0.43, ${\text{η}}_{p}^{2}$ = 0.03]. However, the traditional TSST evoked significantly higher levels of cortisol [*F*(2.32,76.57) = 7.16, *p* = 0.001, ${\text{η}}_{p}^{2}$ = 0.18] compared to the e-TSST condition (see Figure 3). Follow-up contrasts revealed that the traditional TSST produced higher cortisol reactions at all post-stressor assessments [5:15 *t*(39) = 2.09; 5:30 *t*(38) = 4.78; 5:45 *t*(38) = 5.70; 6:15 *t*(37) = 5.52, *p*’s < 0.05].

## Discussion

The primary aim of this study was to develop and validate a laboratory-based electronic model of the TSST by comparing the subjective and biological stress responses of participants in the e-TSST condition to those in the e-neutral condition. To the best of our knowledge, this is the first study to date to employ pre-recorded live actors within an electronic environment in a TSST paradigm. To further assess the effectiveness of the e-TSST, an exploratory aim was to compare the stress response evoked by the e-TSST to the stress response evoked by the traditional TSST, using archival data.

### e-TSST

The efficacy of the e-TSST condition was compared to the e-neutral condition, covarying for baseline levels of stress. The e-TSST condition evoked significant increases among all indices of stress (i.e., SUDS, cortisol, BP), with the exception of HR, when compared to the e-neutral condition. It is important to consider that HR, because of high variability both within and between participants during the laboratory challenge, often fails to be a sensitive index of objective stress response (Jorna, 1992; Quintana and Heathers, 2014). Both SUDs and BP levels evidenced a significant increase at the first post-stressor assessment, followed by a steady decrease throughout the remainder of the session. In contrast, cortisol levels continued to increase over time and were significantly higher at the second post-stressor assessment, and then decreased steadily. Whereas self-report (i.e., SUDs) and BP measurements allow for immediate evaluation of stress reactivity, cortisol must be secreted and enter the vascular system, thus taking longer for the effect to be seen (Eriksson et al., 1998). This process is likely why participants’ SUDs (which were significantly higher at the first two post-stressor assessments) and systolic BP (which was significantly higher at the first post-stressor assessment) levels began to immediately decrease following the initial first post-stressor increase, but cortisol did not show an increase until the second post-stressor assessment.

Our results are mostly closely comparable to those reported by Jönsson et al. (2010), who used mounted headgear to immerse their participants into their VR version of the TSST that utilized computerized confederates. Jönsson et al. (2010) found that the VR-TSST evoked significant increases in cortisol and HR at the first stress provocation when compared to a neutral condition; subjective stress was not assessed in their study. Our study expands the current literature by introducing a widely applicable, low-cost, low burden electronic medium for administering the TSST. Unlike VR-TSST paradigms that utilize immersion headsets, the e-TSST can be used readily in any lab setting. Additionally, this electronic version of the TSST utilizes a medium that has become societal commonplace. With the frequent use of electronic mediums like Skype and Facetime, the e-TSST arguably increases believability more than the use of an immersion headset. Furthermore, this is the first study of its kind to examine the efficacy of a virtual or electronic TSST compared both a control condition as well as to a live TSST paradigm. Thus, studies like Jönsson et al. (2010) that compared cortisol levels between VR-TSST and VR-Neutral conditions may be misleading in their explanation of VR-TSST efficacy, as they are lacking a quality comparison to a widely used and empirically supported live version of the task.

It is important to note that future research studies should assess for multiple stress indices, including subjective self-report measures and biological assessments, as the present findings suggests that the TSST may differentially impact these measures. Similarly, the present findings extend the results found in other studies that demonstrate significant neuroendocrine (Bullinger et al., 2005) and physiological (Grillon et al., 2006) responses elicited by stress tasks administered within a VR environment. Finally, the current findings are also consistent with other studies evidencing the ability of VR paradigms to evoke anger and fear responses (e.g., Rothbaum et al., 2000; Macedonio et al., 2007). Thus, a growing body of research attests to the ability of electronic/VR paradigms to elicit stress and negative emotions.

### e-TSST vs. Traditional TSST

The e-TSST performed equally as well as the traditional TSST on three of the four indices of stress assessed (SUDS, BP, and HR). Although the e-TSST led to higher cortisol response than the e-neutral condition, the traditional TSST evoked higher cortisol responses across the entire session than did the e-TSST. In Dickerson and Kemeny’s (2004) meta analysis of laboratory stressors, they concluded that tasks such as the TSST, which combines socially evaluative, cognitive, and public speaking tasks, are successful in eliciting significant cortisol responses. Although compared to the e-neutral condition the e-TSST elicited a stronger cortisol response, this response was not as high as that in the traditional TSST. These findings are in line with the study conducted by Kelly et al. (2007), who found that the traditional TSST elicited significantly higher cortisol levels compared to both virtual and imaginary versions of the TSST. This attenuated response in the e-TSST may be due to participants’ doubts about the believability of the e-TSST. This question should be addressed in future studies by collecting information about participants’ perceptions of the overall experience (e.g., whether they believed the electronic confederates were actually in the other room, whether they felt they were being socially evaluated), as these factors may account for the differences in cortisol and HR between the studies. Future studies may also investigate differences in social perceptions between VR vs. traditional TSST paradigms (e.g., participants’ sensitivity to the audience’s perceptions of them) to see if these perceptions influence subjective and biological indices of stress. In sum, although our results suggest that the virtual confederates served a similar purpose to live confederates in eliciting stress responses compared to a neutral condition in the TSST paradigm, the magnitude of their effects on stress reactivity were not as strong as those of the live confederates on one of the four measured indices of stress. Therefore, further investigations regarding believability of the virtual confederates as well as investigations of various virtual mediums are warranted to determine whether electronic versions of the TSST can produce comparable elevations in cortisol to those incited by the traditional TSST.

### Implications

The present findings suggest that utilizing an electronic version of the TSST paradigm can be a valuable method for conducting laboratory-based studies of stress reactivity across multiple indices, although it is currently a less effective paradigm than the traditional TSST in evoking cortisol reactivity. Therefore, refinement of the electronic TSST is necessary—the creation of a paradigm comparable to the traditional TSST will require an iterative process of empirical trials and further investigations. That said, the e-TSST offers a more controlled, standardized version of the social stress paradigm that reduces sources of potential error variance. For example, the use of a consistent group of virtual confederates across the study may eliminate the risk of biases based on the audience’s demographic factors (e.g., sex, age, race). Further, the virtual audience affords the researcher greater control over audience members (e.g., facial expressions, vocal inflection) that could alter the stress response. Replication of research results is also made more feasible, given that identical versions of the e-TSST could be shared between researchers. Although animated confederates may prove more cost effective by eliminating the need to schedule and record live actors, utilizing pre-recorded live actors in their live environments for the e-TSST is more comparable to the standard live TSST. Moreover, the recent societal shift toward electronic means of communication (e.g., Skype, Facetime, etc.) increases believability in the e-TSST. For example, being expected to give a work presentation electronically is arguably becoming just as much a part of society as going to a meeting to give an in-person presentation. As noted above, further research assessing the believability of live vs. virtual actors is needed to assess for differences between these audience types; such work will provide valuable information for the direction in which to continue modifications of this paradigm to maximize its utility and feasibility.

Additionally, the implementation of the e-TSST over the traditional TSST increases ease of administration when conducting research by eliminating the need to recruit, schedule and compensate confederates, resulting in a more time and cost-effective methodology. Yet, although the e-TSST was significantly more effective than the e-neutral control in eliciting stress among several subjective and biological indices of stress, the live version produced significantly higher cortisol increases than the e-TSST. Therefore, further investigation of the reliability of a VR version of this paradigm, and its utility in instigating a stress response, is necessary before it is widely adopted in future stress-induction research.

### Limitations

Limitations of the present study include a modest sample size and significant differences between the age, BDI and ASI-3 scores between the e-TSST and traditional TSST study samples, although these variables were controlled for in analyses. Given that both studies used young healthy, predominantly Caucasian, participants, research using the e-TSST in more diverse samples, including clinical populations, is needed. The aforementioned research showing the utility of VR versions of anxiety treatments (e.g., Rothbaum et al., 2000, 2006; Emmelkamp et al., 2002) is promising in this regard. In addition to focusing on participants’ demographics, it may also be of interest to replicate this study manipulating the demographics of the virtual confederates to reduce potential biases related to demographic characteristics (e.g., gender, age, ethnicity). Additionally, future studies may implement the use of continuous measures of BP. Finally, as previously discussed, believability is a potential limitation of the present study and future research should conduct manipulation checks regarding participants’ perceptions of the virtual audience. Notably, these limitations are consistent with extant research, such that within other uses of the electronic and traditional TSST, composition of the confederate panel had not been compared for (e.g., Kelly et al., 2007; Jönsson et al., 2010). To the best of our knowledge, Kelly et al. (2007) conducted a manipulation check to confirm believability in the imagined TSST condition, but no mention of manipulation check for the virtual TSST was made. The use of a computerized version, as opposed to an immersion headset, was chosen to increase believability and familiarity, as well as to increase applicability, reduce researcher cost and burden, and to reduce the risk of dizziness that often accompanies immersion VR. Findings from the present study must be taken in light of these considerations. Manipulating the demographics of the virtual audience in future studies may alleviate some believability concerns.

## Conclusion

This study is the first to compare an electronic version of the TSST that utilized filmed humans, taped in a live environment, instead of avatars or other computerized options. This is the first electronic or virtual application of the TSST that does not use immersion headset technology, but instead uses an accessible, cost-effective, and socially normalized electronic medium. Results comparing the e-TSST vs. e-neutral conditions suggested that the stress manipulation was effective on most indices of stress measured. Further, exploratory analyses comparing the e-TSST to archival data from the traditional TSST suggest comparability between the paradigms, although the traditional TSST outperformed the e-TSST on one index of stress, calling for further modification to increase the reliability of the electronic paradigm. Taken together, these findings provide initial support for the development of electronic versions of the TSST, although further fine-tuning of the e-TSST is warranted prior to broad adoption of this technology.

### Conflict of Interest Statement

The authors declare that the research was conducted in the absence of any commercial or financial relationships that could be construed as a potential conflict of interest.
